# QuickStats

**Published:** 2014-12-19

**Authors:** FROM THE NATIONAL CENTER FOR HEALTH STATISTICS

**Figure:**
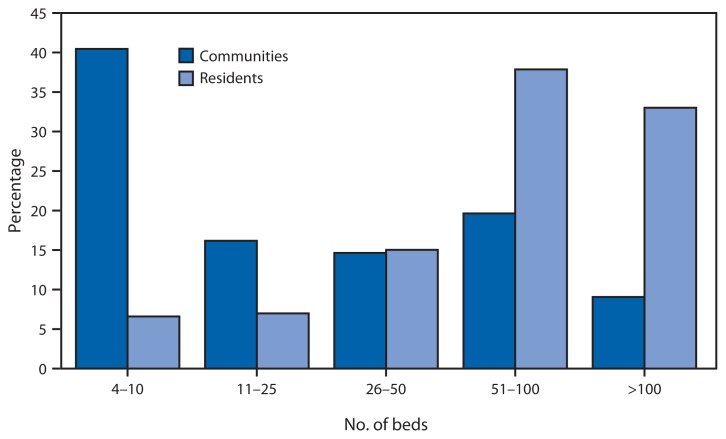
Percentage Distribution of Residential Care Communities* and Residents,^†^ by Number of Beds^§^ — National Study of Long-Term Care Providers, United States, 2012 * Assisted living and similar communities (e.g., personal care homes, adult care homes, board and care homes, and adult foster care). Residential care communities with missing data were excluded. ^†^ Participating administrators and directors of residential care communities were asked, “What is the total number of residents currently living at this residential care community? Include respite care residents.” ^§^ Participating administrators and directors of residential care communities were asked, “At this residential care community, what is the number of licensed, registered, or certified residential care beds? Include both occupied and unoccupied beds.”

In 2012, there were 22,200 residential care communities serving 713,300 residents across the United States. Forty percent of residential care communities were smaller with 4–10 beds, but these communities housed only 7% of all residents. The largest residential care communities with more than 100 beds were only 9% of all communities but housed 33% of all residents.

**Source:** Caffrey C, Harris-Kojetin L, Rome V, Sengupta M. Operating characteristics of residential care communities, by community bed size: United States, 2012. NCHS data brief, no 170. Hyattsville, MD: National Center for Health Statistics; 2014. Available at http://www.cdc.gov/nchs/data/databriefs/db170.htm.

**Reported by:** Vincent Rome, MPH, vrome@cdc.gov, 301-458-4466; Christine Caffrey, PhD; Lauren Harris-Kojetin, PhD.

